# Air Pollution Effects to the Subtype and Severity of Lung Cancers

**DOI:** 10.3389/fmed.2022.835026

**Published:** 2022-03-31

**Authors:** Hung-Chi Lee, Yueh-Hsun Lu, Yen-Lin Huang, Shih Li Huang, Hsiao-Chi Chuang

**Affiliations:** ^1^Department of Radiology, Shuang-Ho Hospital, Taipei Medical University, New Taipei City, Taiwan; ^2^Department of Radiology, School of Medicine, College of Medicine, Taipei Medical University, Taipei, Taiwan; ^3^Medical Department, Tai An Hospital Shuang Shi Branch, Taichung, Taiwan; ^4^School of Respiratory Therapy, College of Medicine, Taipei Medical University, Taipei, Taiwan; ^5^Division of Pulmonary Medicine, Department of Internal Medicine, Shuang Ho Hospital, Taipei Medical University, New Taipei City, Taiwan; ^6^Cell Physiology and Molecular Image Research Center, Wan Fang Hospital, Taipei Medical University, Taipei, Taiwan

**Keywords:** air pollution, lung cancer, staging, adenocarcinoma, squamous cell carcinoma, PM2.5, PM10, NO2

## Abstract

The correlation between lung cancer incidence and air pollution has been established in previous research, but the other detail impact of air pollution to lung cancer is still under investigation. This study aimed to explore if air pollution affected the subtype and staging of lung cancer. At the same time, we investigated the effect of individual pollutant to subtypes and staging. Single center data were extracted from January 1, 2020 to June 30, 2020 using the search engine in the radiology reporting system of Shuang-Ho Hospital, New Taipei City, Taiwan. There were 169 patients finally included for analysis. The nationwide statistics data of lung cancer were extracted from the Taiwan Cancer Registry. The air quality data were extracted from the Taiwan air quality monitoring network. Comparison of the single center lung cancer characteristics with nationwide data was made using the chi-square test. Comparison of the air quality of the living space of the included cases with the average quality in Taiwan in 2020 was made using the *Z*-test. The result shows there was significant difference of cancer subtype and staging between the regional data and the nationwide data. The regional data demonstrated a tendency of higher incidence of adenocarcinoma and advanced stage disease. As for air quality, there was no significant difference. The regional PM10 level presented generally lower levels in regional data as compared to Taiwan in 2020 with near statistically significant *P*-value (0.052); the regional NO_2_ level presented generally higher levels in regional data as compared to Taiwan in 2020 with near statistically significant *P*-value (0.060). The results indicate that air pollution might be related to increase in adenocarcinoma ratio and advanced stage of lung cancer at initial presentation. The NO_2_ was probably the leading pollutant causing this trend.

## Introduction

Lung cancer remains one of the most commonly diagnosed cancer in recent years. With the high incidence, a large number of patients were diagnosed at the late stage of disease, making it the leading cause of overall cancer death (1). To cease the impact of lung cancer, much effort has been made to determine risk factors for prevention (2–11). To date, many risk factors of lung cancer have been identified, including cigarette smoking, radon, asbestos, family history of lung cancer, and air pollution (1). Cigarette smoking, as the leading cause for lung cancer and a modifiable risk factor, has attracted much attention in recent years (12, 13). Following the undertaken measures to prevent the population from smoking, the proportional number of smokers decreased significantly. Recently, air pollution was identified as a risk factor, when in 2013 the International Agency for Research on Cancer (IARC) declared air pollution as a category 1 for human carcinogens (14–24). Being a highly modifiable risk factor of lung cancer, the effect of air pollution on lung cancer gained more attention in recent years. Prior studies identified a correlation between air pollution and cases of lung cancer (25–28). However, there are other studies investigating the effect of air pollution on lung cancer in more detail. These include, for instance, survival rate, type, severity, and the effects of individual pollutants that are still being investigated. In this study we aimed to explore whether air pollution affected the subtype and staging of lung cancer. At the same time, we investigated the effect of individual pollutant to subtypes and staging of lung cancer.

## Materials and Methods

A search engine in the radiology reporting system of Shuang-Ho Hospital, New Taipei City, Taiwan was used for data extraction. The data from January 1, 2020 to June 30, 2020 were included, using the key word “lung cancer(s)” for searching. The search results present a total of 372 imaging studies belonging to 227 patients. The images and clinical history of the 227 patients were checked to exclude not truly diagnosed lung cancer, which appeared in the search machine due to the phrase “lung cancer(s)” that was present in the imaging cases for other reasons. The patients who were clinically diagnosed as having lung cancer, yet without pathologic proof, have also been excluded. A total of 169 patients that were diagnosed as having lung cancer with pathologic proof were considered in this study (Table 1). The staging, pathologic cell type, and living spaces of these patients were documented. As for staging, using pathologic staging as a priority, in case if there was no detected pathologic staging, for instance, a patient with stage IV cancer, the clinical staging was used as a substitute. For patients with recurrent or progression of the disease, or who received neoadjuvant therapy, the staging at initial presentation was used for statistical analysis.

**Table 1:** Demographic characteristics.

Total	169
Age, mean (SD)	66.5 (12)
**Sex (%)**
M	74 (43.8)
F	95 (56.2)
**Tumor location (%)**
Right	106 (62.7)
Left	63 (37.3)
Stage (%)
0	4 (2.4)
1	35 (20.7)
2	8 (4.7)
3	25 (14.8)
4	97 (57.3)
**Tumor type (%)**
Adenocarcinoma	136 (80.4)
Squamous cell carcinoma	23 (13.6)
Other	10 (5.9)

**Figure 1:**
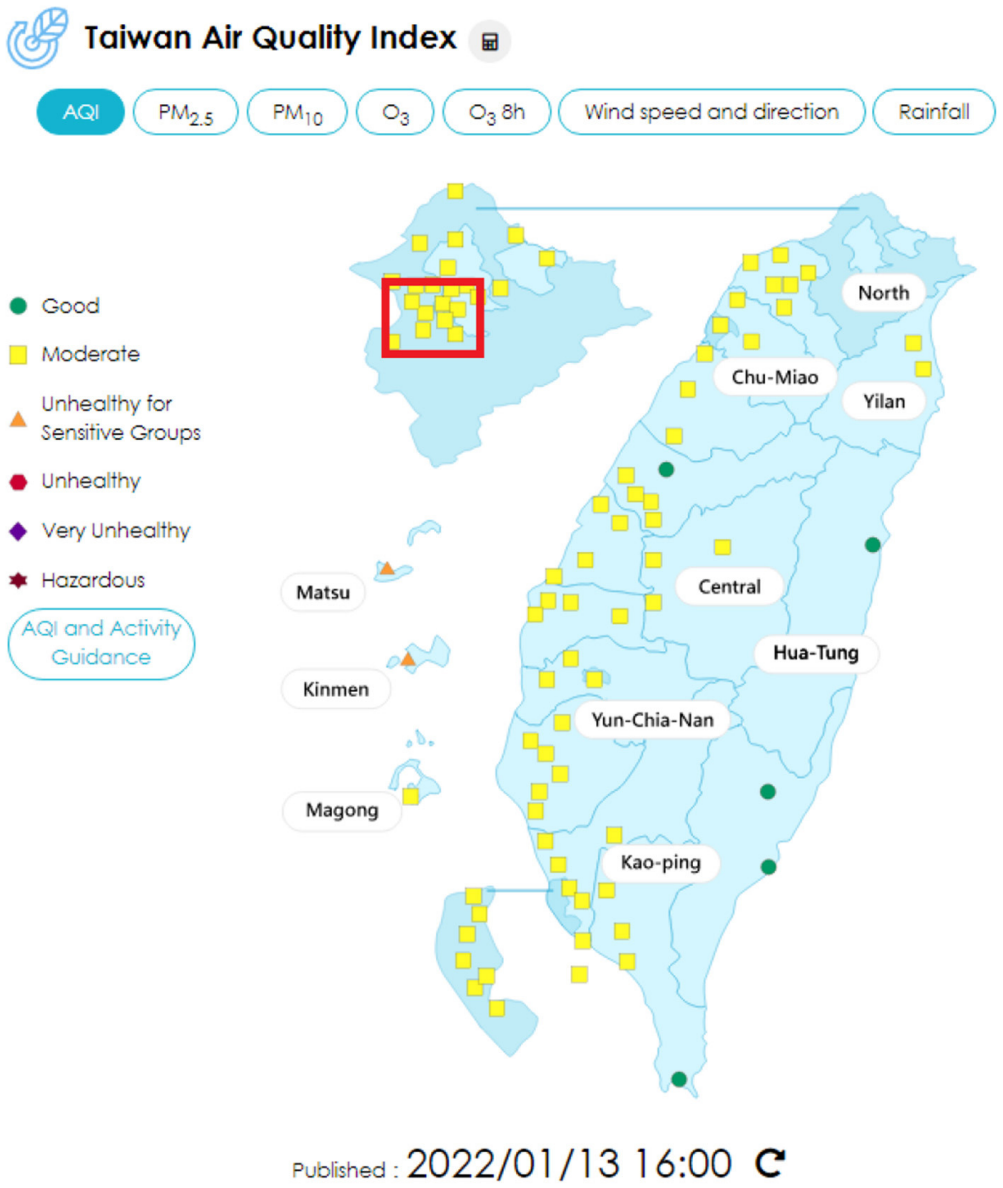
Air quality index distribution in Taiwan, using the data on January 13, 2022 for example. The red block shows the areas involved in this study (29).

**Figure 2:**
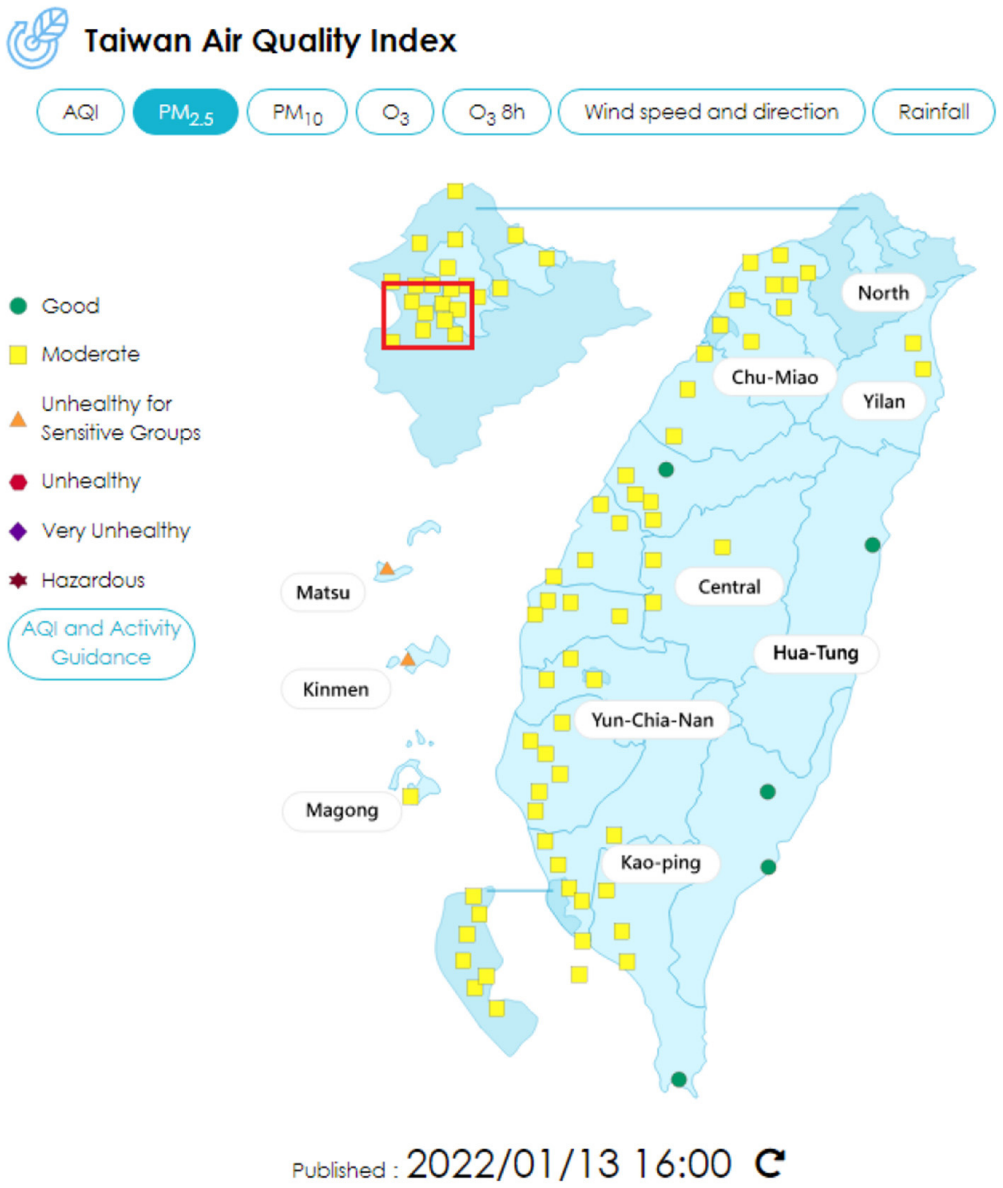
Air quality index of PM2.5 distribution in Taiwan, using the data on January 13, 2022 for example. The red block shows the areas involved in this study (29).

As for data on air quality, the data were extracted from the Taiwan air quality monitoring network, an official website maintained by the Environmental Protection Administration, Executive Yuan, Taiwan (29). The website revealed the real-time data of monitoring stations all over Taiwan, also the yearly statistics data from 1993 to now (Figures 1, 2). The data of 2020 were used for analysis in this study.

The statistical data on lung cancer in Taiwan were accessed from the Taiwan Cancer Registry (30), which is a population-based cancer registry founded in 1979, and operated by the National Public Health Association. At the time of finalizing this study the most recent data were on 2018, which were used for the statistical analysis.

The comparison of the single center lung cancer characteristics with nationwide data was made using the chi-square test. The comparison of the air quality of the living spaces of the 169 included cases with the data of all of Taiwan in 2020 was made using the *Z*-test.

## Results

All 169 cases were sorted by living space of the included patients, into four major neighboring districts of Shuang-Ho Hospital, including Zhonghe/Yonghe (ZY), Banqiao (BQ), Tucheng (TC), and Xinzhuang (XZ). The data of corresponding air quality in the four districts were extracted from the Taiwan Air Quality Monitoring Network (Tables 2–**4**).

**Table 2:** The number and percentage of lung cancer subtype.

	**Adenocarcinoma**	**Squamous**	**Other**	**Total**	***P*-value**	***X* ^2^**
ZY	79 (82.3)	14 (14.6)	3 (3.1)	96	0.002	12.279
BQ	16 (84.2)	1 (5.3)	2 (10.5)	19	0.463	1.54
TC	15 (71.4)	4 (19.0)	2 (9.5)	21	0.485	1.449
XZ	3 (75)	1 (25)	0 (0)	4	0.544	1.217
Other	23 (79.3)	3 (10.3)	3 (10.3)	29	0.623	0.948
Total	136 (80.4)	23 (13.6)	10 (5.9)	169	0.001	13.367
Taiwan 2018	10,988 (71.6)	1,849 (12.0)	2,508 (16.3)	15,345		

As for cell type of lung cancer, the data for all of Taiwan were 71.6% of adenocarcinoma, 12.0% of squamous cell carcinoma, and 16.3% of other cell types. The included 169 cases presented in total 80.4% of adenocarcinoma, 13.6% of squamous cell carcinoma, and 5.9% of other cell types. The ZY district presented 82.3% of adenocarcinoma, 14.6% of squamous cell carcinoma, and 3.1% of other cell types. The BQ district presented 84.2% of adenocarcinoma, 5.3% of squamous cell carcinoma, and 10.5% of other cell types. The TC district presented 71.4% of adenocarcinoma, 19.0% of squamous cell carcinoma, and 9.5% of other cell types. The XZ district presented 75.0% of adenocarcinoma, 25.0% of squamous cell carcinoma, and 0% of other cell types. Comparing the cell type percentage of all 169 cases to all of Taiwan, we found a significant difference (*P* < 0.05). As comparing the four districts' data separately with all of Taiwan, the ZY district presented significant difference (*P* < 0.05), while the other three districts presented no significant difference (*P* > 0.05; Table 2).

As for staging of lung cancer, the Taiwan data in 2018 presented 4.8% of stage 0 disease, 31.4% of stage I disease, 4.2% of stage II disease, 11.6% of stage III disease, and 46.7% of stage IV disease. The included 169 cases presented a total of 2.4% of stage 0 disease, 20.7% of stage I disease, 4.7% of stage II disease, 14.8% of stage III disease, and 57.3% of stage IV disease. The ZY district presented 3.2% of stage 0 disease, 15.8% of stage I disease, 4.7% of stage II disease, 14.8% of stage III disease, and 57.3% of stage IV disease. The BQ district presented 5.3% of stage 0 disease, 26.3% of stage I disease, 5.3% of stage II disease, 5.3% of stage III disease, and 57.9% of stage IV disease. The TC district presented 0% of stage 0 disease, 44.4% of stage I disease, 0% of stage II disease, 27.8% of stage III disease, and 27.8% of stage IV disease. The XZ district presented 0% of stage 0 disease, 25% of stage I disease, 0% of stage II disease, 0% of stage III disease, and 75% of stage IV disease. Comparing the staging percentage of all 169 cases to all of Taiwan, there was a significant difference (*P* < 0.05). As comparing the four-district data separately with all of Taiwan, the ZY district presented significant difference (*P* < 0.05), the other three districts presented no significant difference (*P* > 0.05; Table 3).

**Table 3:** The number and percentage of lung cancer stage.

	**Stage 0**	**Stage 1**	**Stage 2**	**Stage 3**	**Stage 4**	**Total**	***P*-value**	***X* ^2^**
ZY	3 (3.2)	15 (15.8)	6 (6.3)	14 (14.7)	57 (60)	95	0.011	13.0488
BQ	1 (5.3)	5 (26.3)	1 (5.3)	1 (5.3)	11 (57.9)	19	0.852	1.358
TC	0 (0)	8 (44.4)	0 (0)	5 (27.8)	5 (27.8)	18	0.0937	7.941
XZ	0 (0)	1 (25)	0 (0)	0 (0)	3 (75)	4	0.817	1.552
Other	0 (0)	6 (18.2)	1 (3.0)	5 (15.2)	21 (63.6)	33	0.208	5.878
Total	4 (2.4)	35 (20.7)	8 (4.7)	25 (14.8)	97 (57.3)	169	0.008	13.735
Taiwan 2018	656 (4.8)	4,230 (31.4)	577 (4.2)	1,563 (11.6)	6,281 (46.7)	13,444		

As for air quality, the average AQI (air quality index) of all air monitoring stations in Taiwan in 2020 was 57. The ZY district was 40, 48 in the BQ district, 56 in the TC district, and 54 in the XZ district (Table 4). The ambient air pollutants' concentrations include PM10, PM2.5, SO_2_, NO_2_, CO, and O_3_ of all monitoring stations in Taiwan in 2020 as well as the individual data of monitoring stations at ZY, BQ, TC, and XZ districts are demonstrated in Table 5. The overall data of ambient air pollutants' concentrations of the four districts were compared to the overall data of Taiwan using the *Z*-test. The concentration of all pollutants presented no significant difference between the two results. However, the PM10 and NO_2_ presented low *P*-value (0.052 and 0.060, respectively) close to statistical significance. The PM10 presented generally lower levels in the four districts as compared to Taiwan in 2020. The NO_2_ presented generally higher levels in the four districts as compared to Taiwan in 2020 (Table 5).

**Table 4:** Air quality index.

**AQI data**					**0–50**		**51–100**		**101–150**	
**Location**	**Average**	**SD**	**Lowest**	**Highest**	**Days**	**%**	**Days**	**%**	**Days**	**%**
ZY	40	47	7	92	282	77.05	84	22.95	0	0
BQ	48	24	11	182	261	71.31	94	25.68	8	2.19
TC	56	30	12	200	224	61.2	117	31.97	17	4.64
XZ	54	26	13	190	223	60.93	123	33.61	15	4.1
Total Taiwan 2020	57				11,405	54.67	7,397	35.46	1,850	8.87

**Table 5:** The concentration of ambient air pollutants in 2020.

**Location**	**PM10 (μg/m^**3**^)**	**PM2.5 (μg/m^**3**^)**	**SO_**2**_ (ppb)**	**NO_**2**_ (ppb)**	**CO (ppm)**	**O_**3**_ avg (ppb)**
ZY	23	12.2	1.84	15.7	0.63	28.21
BQ	24.4	12.5	2.4	17.35	0.45	26.92
TC	24.1	12.9	1.93	14.03	0.39	30.7
XZ	26.5	13.8	2.6	15.11	0.43	30.49
Taiwan 2020	30.2	15.1	2.13	11.16	0.35	30.9
Taiwan 2020 SD	8.1	3.7	0.55	5.01	0.15	3.95
Regional SD	2.14	0.48	0.13	1.92	0.011	3.35
*P*-value	0.052	0.25	0.93	0.06	0.75	0.41

## Discussion

The data used in this study is from January 2020 to June 2020. There was a worldwide outbreak of COVID-19 infection in 2020, therefore, by shutting the borders early and requiring 2-week quarantines of nearly everyone who arrived from overseas, there was not a pandemic outbreak in Taiwan until May 2021, and most residents did not wear masks until May 2021. Therefore, the influences of COVID-19 infection on the study results are considered minor.

This study demonstrated significant difference between the subtypes of lung cancer in the ZY district and the total four districts when compared to the data of Taiwan in 2020. We detected that the ratio of adenocarcinoma was higher in these areas. At the same time, the PM10 concentration was significantly lower in the ZY district and in all four districts. On the other hand, we noted that the NO_2_ concentration had much higher values. There were previous studies that suggested a certain correlation between air pollution and squamous cell carcinoma (31). However, the majority of studies found that ambient air pollution increased the incidence of adenocarcinoma with little to no effect on squamous cell carcinoma (25, 26, 32–34). As for the specific effect of each air pollutant, many studies reported previously that PM2.5 and PM10 are mostly associated with the increasing risk of adenocarcinoma, whereas the effects of NOx on lung cancer subtype were not sufficiently discussed (25–28, 33). This was somehow a paradoxical result when considering prior studies of biological effect of these substances. Nitrates and toxic agents formed from NOx were thought to be associated with adenocarcinoma formation, whereas the polycyclic aromatic hydrocarbons, one of the primary organic compound clusters in particulate matter, was thought to be associated with squamous cell carcinoma formation (35, 36). In this study, the increasing trend in adenocarcinoma ratio was accompanied with a trend of increase in NO_2_ concentration, and with a decrease in PM10 concentration, a result that was more compatible with the biological effect of these substances, but inconsistent with some prior statistical studies (25, 27). More studies are needed to investigate the definite effect of each pollutant to lung cancer subtype.

The staging of lung cancer in the ZY district and in the total four districts presented significant differences compared to Taiwan in 2020. The ratio of late-stage disease was higher in the ZY district and in the total four districts. Prior study had presented that air pollution exposure shortens lung cancer survival (32). This effect was especially prominent with the early-stage patients and adenocarcinoma. The exposure of PM2.5, PM10, and NO_2_ all presented increased risk of death (32, 34, 37, 38). In our study, the NO_2_ concentration in the ZY district and in all the four districts were higher than in all of Taiwan in 2020 with a significant *P*-value. The PM2.5 presented no significant difference, while the PM10 presented a near significant decrease in the ZY district and in all four districts than Taiwan in 2020. The obtained results point at the possibility that NO_2_ plays a key role in the increasing severity of lung cancer. Assessment of the evidence of NO_2_ had been conducted by the US Environmental Protection Agency Integrated Science Assessment and Health Canada, and came out with the conclusion that the evidence was suggestive of, but not sufficient to infer, a causal relationship between long-term exposure to NO_2_ and mortality among the adults (39). However, the existing reviews are limited by 2014. A more recent meta-analysis including new evidence focused on the effect of NO_2_ presented a positive association between the NO_2_ and the risk of mortality in several types of disease, including lung cancer (37). As a contribution to the existing case studies, the presented result supported the assumption that NO_2_ might be causative to the increase of the mortality in cases of lung cancer by applying and investigating the additional data.

This study had several limitations. First, this was a single center study, not a population study. The ratio of lung cancer subtype and staging in a single center might not be totally compatible with the population ratio. Second, the air quality in the districts analyzed in this study was generally well, as well as in all of Taiwan. The highest AQI was only 56, and Taiwan was only 57, the results of this study might only be applicable in low air pollution areas. This was a study that demonstrated that even in concentrations lower than current EU limit values and below WHO Air Quality Guidelines (27, 40), particulate matter still increased the risk of lung cancer, and there was no threshold. However, the effect other than increasing risk and the effect of other air pollutants in low concentration was not clear. Being a limitation at the same time, this study might give additional information to the effect of air pollutants under low concentration, which was still a poor explored field. Third, the group size of this study was relatively small. Only 169 cases were included for analyzation. The small sample size might cause bias.

## Conclusion

The detail impact of air pollution on lung cancer is still under investigation. This study adds more data to the field and shows that air pollution is related to an increase in the adenocarcinoma ratio and severity of lung cancer at initial presentation. The NO_2_ was probably the leading pollutant causing this trend. However, more research is still needed to justify this correlation due to the inconsistency of results of currently published studies.

## Data Availability Statement

The original contributions presented in the study are included in the article/supplementary material, further inquiries can be directed to the corresponding author/s.

## Ethics Statement

Ethical review and approval was not required for the study of human participants in accordance with the local legislation and institutional requirements. Written informed consent from the patients/participants was not required to participate in this study in accordance with the national legislation and the institutional requirements.

## Author Contributions

H-CL, Y-HL, and H-CC devised the project, the main conceptual ideas. H-CL performed the analytic calculations. All authors contributed to the final version of the manuscript. All authors provided critical feedback and helped shape the research, analysis and manuscript.

## Conflict of Interest

The authors declare that the research was conducted in the absence of any commercial or financial relationships that could be construed as a potential conflict of interest.

## Publisher's Note

All claims expressed in this article are solely those of the authors and do not necessarily represent those of their affiliated organizations, or those of the publisher, the editors and the reviewers. Any product that may be evaluated in this article, or claim that may be made by its manufacturer, is not guaranteed or endorsed by the publisher.
